# Epstein–Barr Virus Genetic Variation in Lymphoblastoid Cell Lines Derived from Kenyan Pediatric Population

**DOI:** 10.1371/journal.pone.0125420

**Published:** 2015-05-01

**Authors:** Kenneth O. Simbiri, Nicholas A. Smith, Richard Otieno, Eric E. M. Wohlford, Ibrahim I. Daud, Sumba P. Odada, Frank Middleton, Rosemary Rochford

**Affiliations:** 1 Department of Microbiology and Immunology, Upstate Medical University, Syracuse, New York, United States of America; 2 Maseno University, Maseno, Kenya; 3 Kenya Medical Research Institute, Kisumu, Kenya; 4 Department of Neuroscience and Physiology, Upstate Medical University, Syracuse, New York, United States of America; Fudan University, CHINA

## Abstract

Epstein-Barr virus (EBV) is associated with Burkitt’s lymphoma (BL), and in regions of sub-Saharan Africa where endemic BL is common, both the EBV Type 1 (EBV-1) and EBV Type 2 strains (EBV-2) are found. Little is known about genetic variation of EBV strains in areas of sub-Saharan Africa. In the present study, spontaneous lymphoblastoid cell lines (LCLs) were generated from samples obtained from Kenya. Polymerase chain reaction (PCR) amplification of the EBV genome was done using multiple primers and sequenced by next-generation sequencing (NGS). Phylogenetic analyses against the published EBV-1 and EBV-2 strains indicated that one sample, LCL10 was closely related to EBV-2, while the remaining 3 LCL samples were more closely related to EBV-1. Moreover, single nucleotide polymorphism (SNP) analyses showed clustering of LCL variants. We further show by analysis of EBNA-1, BLLF1, BPLF1, and BRRF2 that latent genes are less conserved than lytic genes in these LCLs from a single geographic region. In this study we have shown that NGS is highly useful for deciphering detailed inter and intra-variations in EBV genomes and that within a geographic region different EBV genetic variations can co-exist, the implications of which warrant further investigation. The findings will enhance our understanding of potential pathogenic variants critical to the development and maintenance of EBV-associated malignancies.

## Introduction

Epstein-Barr virus (EBV) is a ubiquitous human gammaherpesvirus that infects more than 95% of the human population and has been associated with malignant diseases such as Burkitt’s lymphoma (BL), nasopharyngeal carcinoma (NPC), Hodgkin’s disease, B and T-cell lymphomas, and nonmalignant diseases such as infectious mononucleosis [1]. There are two major strains of EBV: EBV Type 1 (EBV-1) and Type 2 (EBV-2), that have significant genetic differences in the EBV latent genes, Epstein-Barr nuclear antigen (EBNA)-2, 3A and 3C [2–4]. A number of studies have analyzed genetic variability of the different EBV strains based on specific EBV genes such as LMP-1 [5, 6], LMP-2A [7], BZLF-1 [8], EBNA-1 [7, 9, 10], EBNA-2 [11], and EBNA-3A, -3B, and -3C [12]. Interestingly, some studies analyzing multiple genes suggest that recombination between strains can occur, further increasing the difficulty of typing EBV viral strains [13–15]. A long-standing question in the field is whether there are genetic variants of EBV that are associated with malignancies, but given our limited understanding of the diversity of EBV, this question remains unanswered [1].

The EBV genome comprises approximately 171kb with about 86 open reading frames. The EBV genome contains unique regions interspersed by four major internal repeats (IR1-IR4) and terminal repeats (TR). Within the unique region are encoded nine latent proteins including EBNA-1, -2, -3A, -3B, -3C, and-LP, and latent membrane protein 1(LMP-1), LMP-2A, and LMP-2B. Lytic proteins, transcription factors, and capsid proteins are encoded in other open reading frames (ORFs) as well as non-coding RNAs such as Epstein—Barr virus-encoded small RNA1 (EBER1) and EBER2 [13]. With the advent of next generation sequencing (NGS) technology, the opportunity to identify EBV genetic diversity across the genome is being realized [16–18]. A challenge however for sequencing the entire genome is the difficulty in sequencing through the repeat regions. In addition, having adequate viral DNA from peripheral blood samples is also a challenge. As an alternative approach, in this study we utilized a PCR-amplification based approach [17] to sequence all of the ORFs and non-repeat regions covering more than 65% of the EBV genome from lymphoblastoid cell lines (LCL) spontaneously derived from peripheral blood lymphocytes isolated from Kenyan children.

## Materials and Methods

Peripheral blood mononuclear cell (PBMC) samples cultured in the presence of cyclosporine were used to derive four spontaneous LCLs. PBMC were isolated from Kenyan children from a previously described cohort [19]. The Kenya Medical Research Institute (KEMRI) Ethical Review Committee and the Institutional Review Board at State University of New York Upstate Medical University gave the ethical approval for the study. The written consent form was signed by the guardian. Four LCLs were generated from four individual PBMC samples: LCL1, LCL3, LCL9, and LCL10. The LCLs along with two other cell lines, B95.8 (a gift from George Miller, Yale University) [7, 20] and Jijoye (a gift from John Sixbey, LSU Health Sciences Center) [21] were maintained in RPMI-1640 medium with 10% fetal bovine serum (FBS) at 37°C in 5% CO_2_ until DNA extraction. EBV-1 and EBV-2 were subtyped using EBNA3C conventional PCR and electrophoresis (Forward primer: 5’- AGA AGG GGA GCG TGT GTT G -3’ and Reverse primer: 5’- GGC TCG TTT TTG ACG TCG G -3’) to generate an EBV-1 product size of 153bp and EBV-2 product size of 246bp [22].

### DNA extraction and sample preparation

DNA was extracted from LCL1, LCL3, LCL9, LCL10, B95.8, and Jijoye cell lines. PCR was performed on the samples using multiple primer sets designed to cover more than 65% of the non-repetitive EBV genome, including all ORFs [17]. A total of 59 distinct amplicons were generated with these primers (Table 1) using standard cycling conditions. It is to be noted that though most primer sets targeted single gene regions, there is overlap of some genes, and multiple genes covered by single primer sets. Further, in some cases the same primer pairs used covered different regions of EBV-1 and EBV-2. Briefy, 50 ng of DNA were used in 25 μl reaction mixture and run for 35 cycles at 94°C denaturation for 45 seconds, annealing at 56°C for 45seconds, and extension at 72°C for 2 to 6 minutes. Internal and terminal repeats were excluded from the PCR amplification. The PCR products were then purified by QIA PCR purification kit (Qiagen) and equimolar concentrations per amplicon from each subject were pooled together to make 6 sequencing libraries using a single index per subject or control strain. The Illumina Nextera XT kit (San Diego, CA) was used in library construction and all six indexed libraries were combined into a single multiplexed sample for sequencing. The entire process was completed in two runs, with the first run encompassing the first 35 primers and the second run the remaining set of primers.

**Table 1:** Primer sequences used in PCR to amplify LCL samples against EBV-1/ EBV-2.

Seq #	Primer Pair	Product size	Product coordinates	Genes covered-EBV1/ EBV-2
1	TTCTGGTGATGCTTGTGCTC	2076	540–2616	BNRF1
	TGCTGGCGTCTCATAAACAG			
2	CTGTTTATGAGACGCCAGCA	4764	597–7361	BNRF1
	TTTTCGCTGCTTGTCCTTTT			
3	CCTGTGTGACCCCTCACTTT	4567	5545–10112	BNFRF1, EBER1/2, BCRF1, EBNA1
	TCCTTTTTCCTGCAGCTTGT			
4	AAAAGGACAAGCAGCGAAAA	4307	7342–11649	BSLF1, BSRF1, BSLF2
	GTGCAGGAGGCTGTTTCTTC			
5	TTATGGTTCAGTGCGTCGAG	4007	10971–14978	EBNA1, EBNA3A, EBNA2
	GAACTGAGGAGGGCATGAAG			
6	ATGCCTACATTCTATCTTGCGTTAC	1439	36216–37655	EBNA2, EBNA3A, EBNA3B/C, EBNA-LP
	TTACTGGATGGAGGGGCGAGGTCTT			
7	AGGGATGCCTGGACACAAGA	1412	36522–37934	EBNA2
	AACATGGACTGGGAGTGGAG			
8	CTAGAGGTCCGCGAGATTTG	1251	40696–41947	BHRF1
	AGAAGGCAAGCGAAAATTGA			
9	GCAGGCAGTACGAGATGTCA	2239	41700–43939	BHRF1
	TCCCTTCACATCCCAGAGAC			
10	CGACATTGACAGCCTTCTCA AAACACGAATGCCAAGAACC	4312	43795–48107	BFLF1, BFRF1A, BFRF2
11	TGCTCCTGATGTTTCTGAGGTGGA	1776	47586–49362	BFRF3
	AGGTAACTTCTTTGAGCCTCCCGA			
12	TTGCTCCATCTGTCAGCAAC	1759	49088–50847	BOLF1
	CACAAGCCTCCTCTCAGGAC			
13	GGTGACCACTGAGGGAGTGT	1789	50045–51834	EBNA1
	CTTTCGAGCCAGAGATGTCC			
14	CCGAAATAGGGCCTTGCCATCAAT	1046	51199–52245	EBNA1
	ATTTCAGGACTACCTGCGCGACTT			
15	GGACATCTCTGGCTCGAAAG	4689	51815–56504	EBNA1, BOLF1, BPLF1
	AGGAGGAGAACCCGAGGATA			
16	TCAGGAGGTCGTCAAAATCC	1720	56125–57845	EBNA1
	AGTAATCCCCATCCCTCACC			
17	TCCAGGCTGTTGGAGAACACTTCA	1172	57133–58305	EBNA1
	TTTCACATCCGACTCATTCCCTGC			
18	GGTGAGGGATGGGGATTACT	2035	57826–59861	EBNA1
	ATCACAGTCACCCCCAGAAG			
19	GCTCATATACGCCACCGTCT	1768	59222–60990	EBNA1
	GCTCATATACGCCACCGTCT			
20	CAGACGGTGGCGTATATGAG	4019	60970–64989	BOLF1, BORF1, BOLF2, EBNA1, BORF2
	CAAAGAGCCCCGTAAAGATG			
21	AACAGGCGGGCGAATGTGTAAT	1272	60125–61397	EBNA1
	ACCTTTCATCCGAACTCCTCAGGT			
22	GCCTCTATGTCGCTCTGACC	4771	63737–68508	BARF1, BORF2, BMRF1
	CGGAGGCGTGGTTAAATAAA			
23	GCGAGCCATAAAGCAGTTTC	4647	67334–71981	BMRF1, BMRF2, BSLF1, BMLF1
	TCTCCCGAACTAGCAGCATT			
24	CTCGCGTGTTAGGAAGGAAG	5649	70814–76463	BSLF1/2, BMLF1, BSRF1, BLLF3
	AGGCAAAGCTGGTCAAAGAA			
25	AGAAGCGCCGGTACTTGTTAAGGA	1495	75554–77049	BLLF3, BLRF1, BLRF2
	TTGATTCTCGTGGTCGTGTTCCCT			
26	GCCTTCTTTGACCAGCTTTG	5215	76441–81656	BLRF1/2, BLLF1
	GACGGGTTCTACTGGCATGT			
27	GGTGAAACGCGAGAAGAAAG	4797	81084–85881	EBNA3A, EBNA3B, EBNA3C
	TTTAGCAGTTCCTCCGCACT			
28	CCCCATCAGACACCTCAAGT	2138	85093–87231	EBNA3B/C
	CACCTCCCGTTGCTAACATT			
29	AGTGCGGAGGAACTGCTAAA	2917	85861–88778	EBNA3B/C
	TGCAGAGGATGAGACCAGTG			
30	CCCACCACGTCTTCAACTTT	2088	88730–90818	BZLF1
	CCATACCAGGTGCCTTTTGT			
31	TCCAAGGTGACCCCTGTTAG	4859	89141–94000	BZLF2, EBNA3B, EBNA3C, BRLF1
	TGATGCAGAGTCGCCTAATG			
32	ACTCCCGGCTGTAAATTCCT	4923	92774–97697	BRRF1, BRRF2, BRLF1, EBNA1
	TGGCCAGAAATACACCAACA			
33	GTGATGAGGACGAGGATGGT	4100	95485–99585	BKRF2, BKRF3, BKRF4
	TCGTGGATGCCCTAAAGAAC			
34	CCCATGTTGTCACGTCACTC	5281	97747–103028	BBLF4, BBRF1
	CACCGTGTTGGAGACCTTTT			
34	ACAGACCATCTACGCCAACC	5392	102044–107436	BBRF1, BBRF2, BBRF3
	CCACCACAAGAAGGTGTCCT			
36	TACGGGGCACTTAACCTGAC	4005	107002–111007	BBRF3, BGLF3, BGLF3.5, BGLF4, BGLF5, BBLF1
	TGACGGAGCTGTATCACGAG			
37	GATGTTGCTGGGGCTAATGT	4358	110724–115082	BGLF4, BGLF5, BGLF3, BGLF3.5, BGRF1/BDRF1
	AGAGAGGGAGTTTCGCTTCC			
38	GGCACCATAGCATGTCACAC	4571	113938–118509	BGRF1/BDRF1, BDLF3.5, BDLF4, BGLF1, BGLF4, BGLF2, BDLF1, BDLF3
	AGTCCCAACAACTTCCAACG			
39	AACACCATCCAGCTCTCCTTCGAT	1526	117867–119393	BGRF1/BDRF1, BGLF3.5, BDLF1, BDLF2, BDLF3
	ATGGGTGTCCGACCAATCCATTCT			
40	CGTTGGAAGTTGTTGGGACT CATTTTACCAGGGACGAGGA	4144	118490–122634	BDLF1, BDLF2, BDLF3, BcLF1
41	ATGCACCTCAAAGGTTACCG	4589	121912–126501	BcLF1, BcRF1
	TTGCAAACTCGCATCTTCAC			
42	CCCGTTCACCAAAACAGTCT	4445	122398–126843	BcRF1
	AACCAGGACACGTTGAGACC			
43	GGTCTCAACGTGTCCTGGTT	4287	126824–131111	BTRF1, BXLF1, BXLF2
	GTGAAGGTATGTGCCGGTCT			
44	ACCTCCCATAGCAACACCAG	4195	130704–134899	BXLF1, BXLF2, BXRF1
	CCCGTGCGATGAGTTTATTT			
45	CCTGAGAACGCTCCAGGTAG	4136	133032–137168	BXRF1, BVRF1, BVLF1, BVRF2
	CCTGGTGAGAAGTTGGTGGT			
46	CCAGACATACCCCAAACCAC	4360	136001–140361	BdRF1, BILF2, RPMS1
	CTCCAGAGGGCAGACGTTAG			
47	GCCCGTTGGGTTACATTAAGGTGT	1437	143534–144971	LF3, Repeat region IR4
	CATGCAGTGGTGTCAGACAGGAAA			
48	TTTGGGATGCATCACTTTGA	3279	144203–147482	Repeat region “DR”
	CCTCAAAGGTGTGGTCGTTT			
49	CTTTGGGTTCCATTGTGTGCCCTT	2838	147061–149899	“RPMS1”
	ACCTGGTACATTGTGCCCATCAGA			
50	TCGTGGCTCGTACAGACGATTGTT	3411	147344–150755	“RPMS1”
	TTTGCGCCTTCTCCTGGTTTATGC			
51	CCCACACCTTCACTCCTTGT	4112	148290–152402	BILF1, LF1, LF2, BALF5
	CAGAGCCAGGCACATCTACA			
52	CCCACACCTTCACTCCTTGT	4002	151117–155119	A73
	CAGAGCCAGGCACATCTACA			
53	TAGTAGCGGGCAACGAGAGA	4030	151842–155872	A73
	CGTGTGTGTGAACGTGTTTG			
54	TGGAAGAAGGCGTAGAGCAT	4181	155195–159376	A73
	CTTGTTTACCCAGACCCTGA			
55	ACGCCGAGTCATCTCTCATTTGGA	1080	158854–159934	A73, BALF3, BALF4
	GCAAGGCTGACTCACCTGTTTGA			
56	GCTCAGGGTCTGGGTAAACA	2368	159355–161723	BARF0, BALF2
	CGTGACTACCCCCACGTACT			
57	AGGTTGCACACCACATCAAA	3871	161261–165132	BALF2, BALF1, BARF1
	GACTCGCTCACCCAAGAAAG			
58	GTGCAGAGCCTTGACATTGA	4183	164101–168284	BALF1, LMP1
	TGAACACCACCACGATGACT			
59	CACGGGGTTTATGTTTCTGG	4002	165676–169678	BRF1, LMP1
	CCCCCTCCACTTTTTCCA			

Note that although most primer sets targeted single gene regions, there is overlap of some genes, and multiple genes covered by single primer sets. Also note that the same primer pairs covered different regions of EBV-1 and EBV-2 in some cases.

### Next generation Illumina sequencing

Both of the multiplexed libraries were sequenced on an Illumina MiSeq instrument using 2x150 paired-end reads (300bp) with the Illumina v2 reagent kit. The two sequencing runs on the multiplexed pooled libraries performed similarly. Initial cluster QC indicated high density (958–1158 clusters/mm^2^) with 86–91% passing QC filter. Total paired-end reads generated were 1.26–1.45 M, with 95.1–95.7% passing a Q30 filter, for a total yield of 346–373 million bp. The. fastq reads were de-multiplexed and indicated approximately equal representation of all samples within each pooled library. Average read depth for each sample was approximately 350 per base across the EBV-1 and EBV-2 genomes (Table 2).

**Table 2:** Alignment statistics for EBV-1 and EBV-2.

Alignment statistics vs. EBV-1						
	B95.8	Jijoye	LCL1	LCL3	LCL9	LCL10
Total number of raw reads	462,896	454,186	389,274	335,880	564,928	542,650
Aligned reads	97.3%	89.9%	95.2%	96.1%	92.9%	92.7%
- Uniquely matched reads	97.1%	89.5%	94.9%	95.5%	92.5%	91.8%
- Number of uniquely aligned reads	449,472	406,496	369,421	320,765	522,558	498,153
Unaligned reads	2.7%	10.1%	4.8%	3.9%	7.1%	7.3%
Average read length	146	142	144	143	144	144
Total Aligned Throughput (Mb)	65.6	57.7	53.2	45.9	75.2	71.7
Average Coverage	382	336	310	267	438	417
Alignment statistics vs. EBV-2						
Total number of reads	462,896	454,186	389,274	335,880	564,928	542,650
Aligned reads	91.9%	95.3%	93.1%	85.9%	87.7%	95.6%
- Uniquely matched reads	91.8%	94.8%	92.7%	85.5%	87.3%	94.6%
- Number of uniquely aligned reads	424,939	430,568	360,857	287,177	493,182	513,347
Unaligned reads	8.1%	4.7%	6.9%	14.1%	12.3%	4.4%
Average read length	146	142	144	144	144	143
Total Aligned Throughput (Mb)	62.0	61.1	52.0	41.4	71.0	73.4
Average Coverage	359	354	301	239	411	425

QC performance against EBV-1 showed a robust performance with high raw reads, high aligned reads, uniform average length read for each sample, and a normal average coverage for the samples. In QC performance against EBV-2 we also observed similar QC performance on alignment against EBV-2 with high raw reads, high aligned reads, uniform average length read for each sample, and a normal average coverage for the samples.

Raw.fastq sequence data were imported directly into Strand NGS 2.0 software for all analyses. Reads were filtered at a Q30 phred score (>95% met this criterion), trimmed to remove any low quality bases, and combined into single sets of paired end reads for each sample. These filtered reads were then aligned to the March 2010 NCBI builds of the EBV-1 and EBV-2 genomes (NC-007605 and NC-009334 respectively) using a minimum 95% matching sequence identity, 5% maximum gaps, 300 bp mean insert length +/- 10 bp, and 50 bp minimum output match length. A comprehensive analysis of sequence differences for each of the six samples compared to each of the two reference genome sequences was then performed within Strand 2.0 software. Single and multi-nucleotide differences were reported only when the sequence coverage exceeded 30 reads at a particular base, the variant sequence appeared in more than 30% of the reads that covered that base, and the-log of the variant score P value exceeded 50. The sequence variants were then mapped to the annotated portions of the genome and classified first by location (intergenic, genic non-coding, genic coding) and type (insertion, deletion, substitution), and then for any effects on amino acids (synonymous, non-synonymous, frameshift).

### Phylogenetic analysis

LCL sample alignment and subsequent analyses were performed using Strand 2.0 software (Strand) on all the 1,243,562 passed reads. Alignment was performed against EBV-1 and EBV-2 sequence (NC-007605 and NC-009334.1 respectively).

## Results

### Summary of sequencing data

Our aim in this study was to determine the genetic variation in EBV genomes based on alignment to the two EBV strains, EBV-1 and EBV-2, and to assess genetic differences that may be significant to EBV pathogenesis. We isolated four LCLs that were spontaneously derived from PBMC from Kenyan children. One of these LCLs, LCL10 derived from a Kenyan child from a malaria holoendemic area was different from the other LCLs that were from malaria hypoendemic region and was identified as having a EBV Type 2 (EBV-2) genome based on PCR amplification of the EBNA3C region of the genome [16]. The other three LCLs were identified as EBV-1 strain. As a reference and control, we included sequencing of the B95.8 virus, which has been used as the reference strain for EBV-1 [7, 20]. We also sequenced the Jijoye cell line, which contains EBV-2 and was derived from a BL patient in West Africa [21]. Following validation and verification of the sequencing runs, we aligned the sequences to EBV-1 (B95.8) and EBV-2 (AG876) genome reference sequences. The LCL reads that mapped onto EBV-1 reference were 462,896 (97.3%) for B95.8, 454,186 (89.9%) for Jijoye, 389,274 (95.2%) for LCL1, 335,880 (96.1%) for LCL3, 564,928 (92.9%) for LCL9, and 542,650 (92.7%) for LCL10. The coverage was 382, 336, 310, 267 438, and 417 respectively. Similarly, the reads that mapped onto EBV-2 reference were 462, 896 (91.9%) for B95.8, 454,186 (95.3%) for Jijoye, 389,274 (93.1%) for LCL1, 335,880 (85.9%) for LCL3, 564,928 (87.7%) for LCL9, and 542,650 (95.6%) for LCL10. The coverage was 359, 354, 301, 239, 411, 425 respectively (Table 2). We observed minimal sequence difference for B95.8 control cell line relative to the published reference sequence (Table 2). The differential alignment results of LCL10 to both EBV-1 and EBV-2 could be due to viral recombination, host-parasite coevolution in the population, or other genetic modifications as a consequence of selective pressure occurring in the region.

### LCL comparative analysis

**Fig 1:**
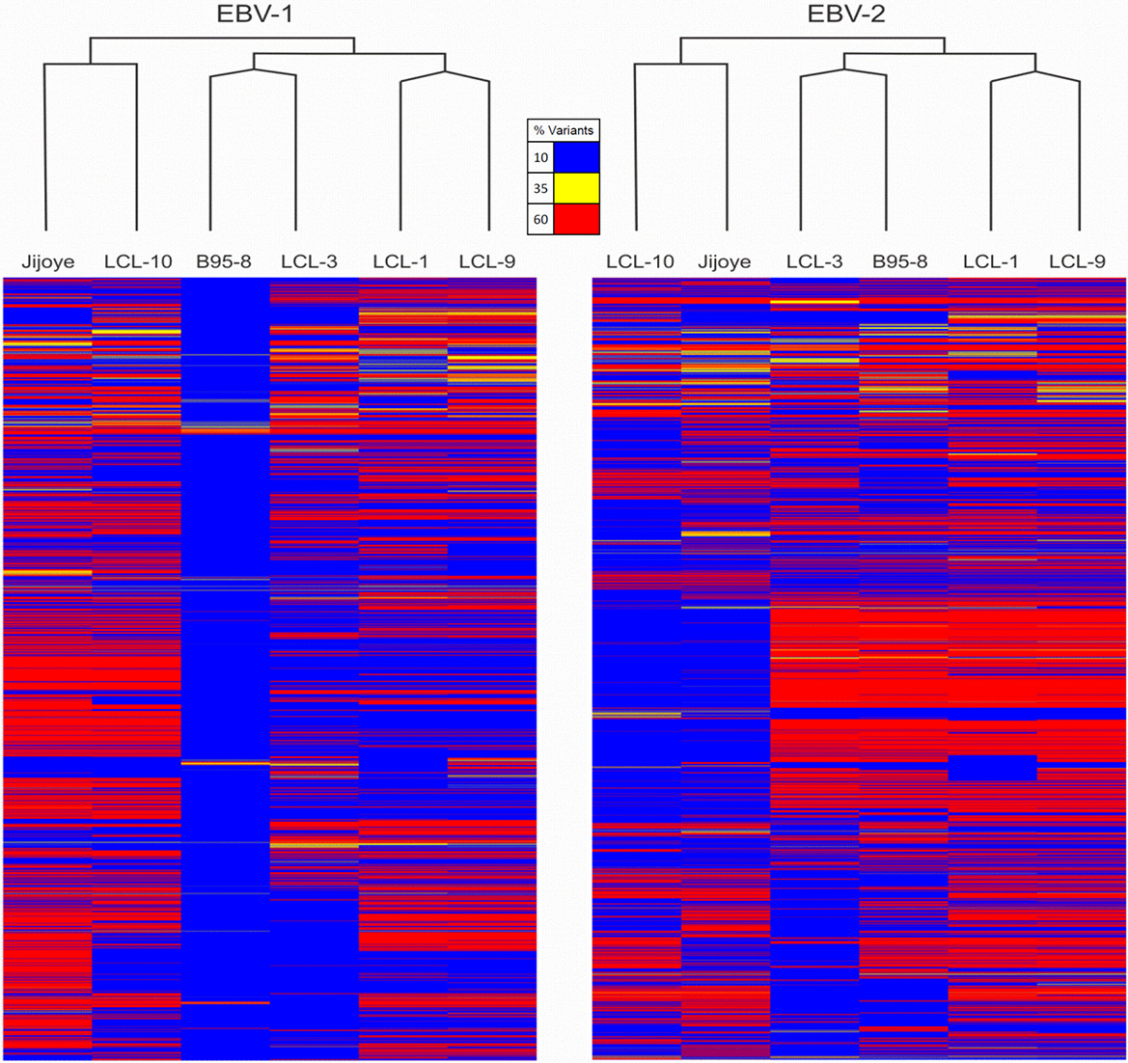
Phylogenetic alignments of LCLs to EBV-1 and EBV-2. The figure shows the alignment of the LCLs, B95.8 cell line and Jijoye cell line controls against EBV-1 and EBV-2. It is observed that B95.8 aligned closely to EBV-1, followed by LCL-3, LCL1 and LCL9, while LCL10 and Jijoye were distant from EBV-1. B95.8 is clustered with LCL3, LCLI and LCL9 are clustered together, while LCL10 and Jijoye are clustered together when compared to EBV-1. There were relatively less base changes in the middle for most of the LCLs except LCL10. Similar trend was observed on comparison with EBV-2, however, LCL10 was much closer to EBV-2 reference than Jijoye and the clustering was maintained as with EBV-1 reference. The bases were more conserved in the middle region of the genome than in the N and C terminus for LCL10 and Jijoye, for B95.8, LCL1, LCL3, and LCL9 the base changes were spread all over the genome with majority of the changes in the middle. Additionally, LCL3 was more conserved at the C-terminal end of the genome, as observed with EBV-1.

We performed phylogenetic analyses on the four LCLs using complete viral genomes from B95.8 and Jijoye cell lines, and EBV-1 and EBV-2 reference genomes (NC-007605 and NC-009334 respectively). Comparisons in non-synonymous and synonymous distributions were made. From the list of SNPs generated for LCLs against EBV-1 and EBV-2, comparisons were made within LCLs to determine similarities and differences among them. Non-synonymous mutations identified in the samples were thereafter categorized by the known and putative functions and ORF designations. When compared to EBV-1, we identified the following number of non-synonymous SNPs: 87 for LCL1, 52 for LCL3, 92 for LCL9, 65 for LCL10, 16 for B95.8, and 108 for Jijoye. It was observed that LCL1, LCL3 and LCL9 were closer to EBV-1 than LCL10 and Jijoye. Similarly, when compared to EBV-2 non-synonymous SNPs were: 137 for LCL1, 125 for LCL3, 130 for LCL9, 50 for LCL10, 115 for B95.8, and 81 for Jijoye. We observed that LCL10 and Jijoye were closer to EBV-2 than the other LCLs and B95.8 (Table 3). Interestingly LCL10 was much closer to EBV-2 than Jijoye as noted in the phylogenetic grouping (Fig 1).

**Table 3:** Summary of all variants in LCL, B95.8, and Jijoye samples compared to EBV-1 and EBV-2.

Variants vs. EBV-1								
Sample	Total	Insertion	Deletion	Substitution	Intergenic	Genic Noncoding	Coding Synonymous	Coding Non-Synonymous
LCL1	968	19	20	929	511	232	136	87
LCL3	606	14	10	582	327	167	59	52
LCL9	969	22	22	925	496	242	137	92
LCL10	980	14	20	946	576	227	110	65
Common LCLs	178	2	0	176	77	56	24	20
B95.8	73	5	5	63	39	8	10	16
Jijoye	1234	13	24	1197	710	215	176	108
Common LCLs+ B95.8+Jijoye	5	0	0	5	4	0	0	1
Variants vs. EBV-2								
LCL1	645	9	9	627	115	226	167	137
LCL3	597	8	11	578	96	226	150	125
LCL9	639	10	9	620	113	238	158	130
LCL10	316	5	9	301	57	117	88	53
B95.8	530	6	10	514	79	206	121	115
Jijoye	423	3	5	415	90	129	123	81
Common LCLs 1,3,9	418	5	7	406	65	166	91	96
Common LCL10+ Jijoye	176	1	2	173	20	50	69	37
Common LCLs 1,3,9+ B95.8	317	3	4	310	43	139	66	69
Common ALL LCLs	85	0	2	83	15	18	28	24
Common ALL samples	49	0	0	49	7	15	16	11

Summary of the mutations identified in the LCLs and control samples when compared to EBV-1, we noted that substitution was the most common type mutation, with Jijoye having the most and B95.8 having the least. Deletions and insertions were also observed ranging from 5 (B95.8) to 24 (Jijoye) and 5 (B95.8) and 13 (Jijoye) respectively. When compared to EBV-2 it is to be noted that substitution was the most common type of mutation, with LCL1 (627) having the most and LCL10 (301) having the least. Deletions and insertions were also observed ranging from 5 (Jijoye) to 11 (LCL-3) for deletion and 3 (Jijoye) to 10 (LCL-9) for insertion respectively.

### Three of the LCLs were phylogenetically closer to each other

We observed that when compared to EBV-1 based on sequence alignment to the reference genomes, LCL1, LCL3, and LCL9 were grouped together while Jijoye and LCL10 were paired together. Further, we observed that LCL3 was much closer to EBV- 1 than LCL1 and LCL9. As expected, the B95.8 cell line control did not differ significantly from the EBV-1 reference (Fig 1). When comparison was made against EBV-2 (AG876 control reference), the LCL10 was highly aligned to EBV-2 than the EBV-2 control cell line Jijoye (Fig 1). We noted less base changes in the center region of the genome when samples were aligned to EBV-1. However, when compared to EBV-2 we observed that the nucleotides were more conserved in the middle of the genome than at the N and C terminus for LCL10 and Jijoye, while the base changes were more spread out in the genome with a majority of the changes in the middle for the LCLs that aligned closely with EBV-1. B95.8 control cell line was very close to EBV-1 reference B95.8, compared to LCL1, LCL3, and LCL9 (Fig 1).

### LCL10 was phylogenetically closer to EBV2 and Jijoye than the other LCLs, but varied in specific latent and lytic genes

**Fig 2:**
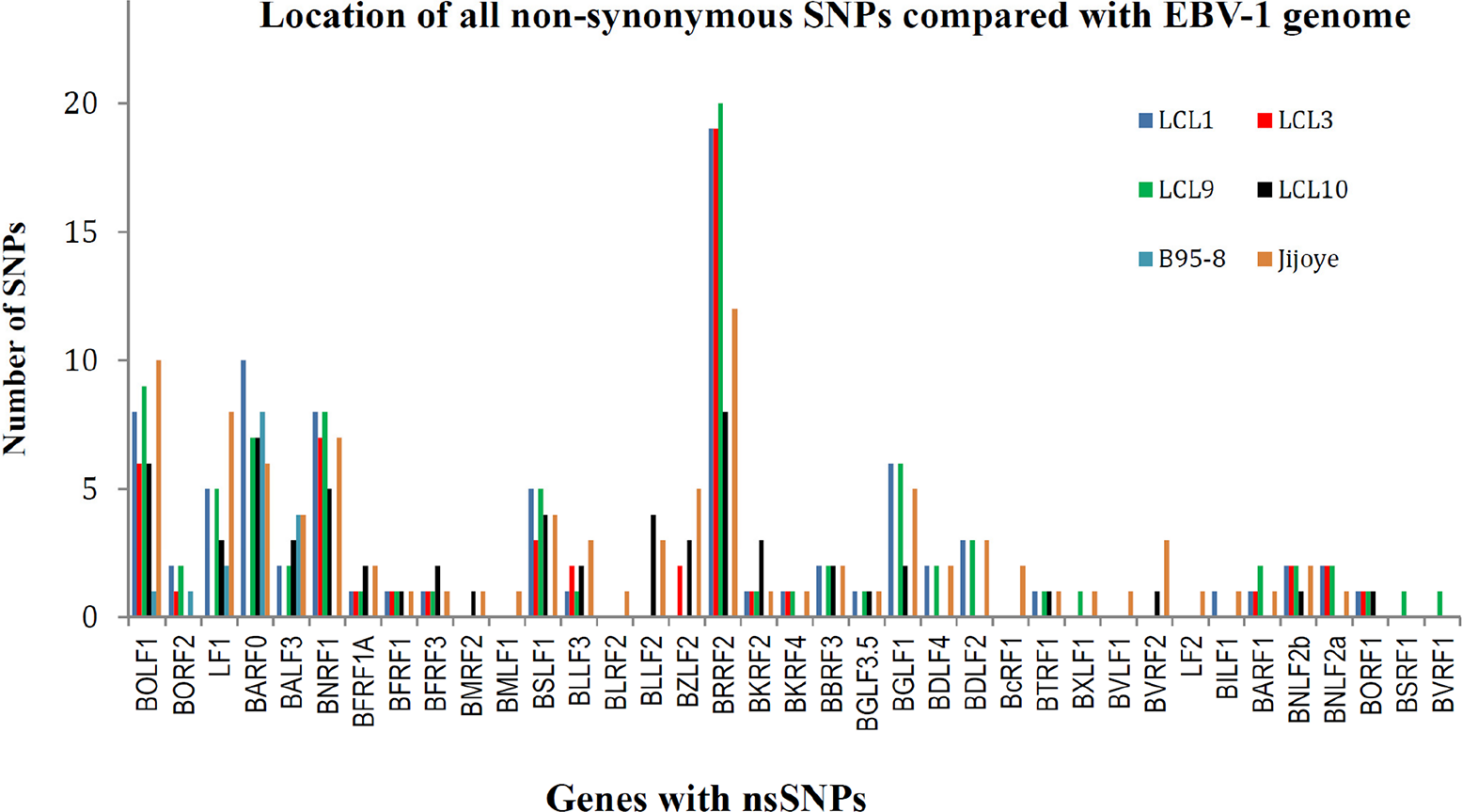
Non—synonymous SNPs compared with EBV-1. When we aligned the sample sequences against EBV-1 reference gene, we observed that BOLF1, BARF0, BNRF1, BSLF1, and BRRF2 were the genes with the most SNPs. BOLF1 had most SNPs in Jijoye and LCL9; BARF0 with most SNPs in LCL1 and B95.8; BNRF1 with most SNPs in LCL3, LCL9 and Jijoye; BSLF1 with more SNPs in LCL1, LCL9 and Jijoye; and BRRF2 had the most number of non—synonymous SNPs in LCL1, LCL3, LCL9, and Jijoye. We observed that all the genes were lytic and mostly tegument and envelop.

**Fig 3:**
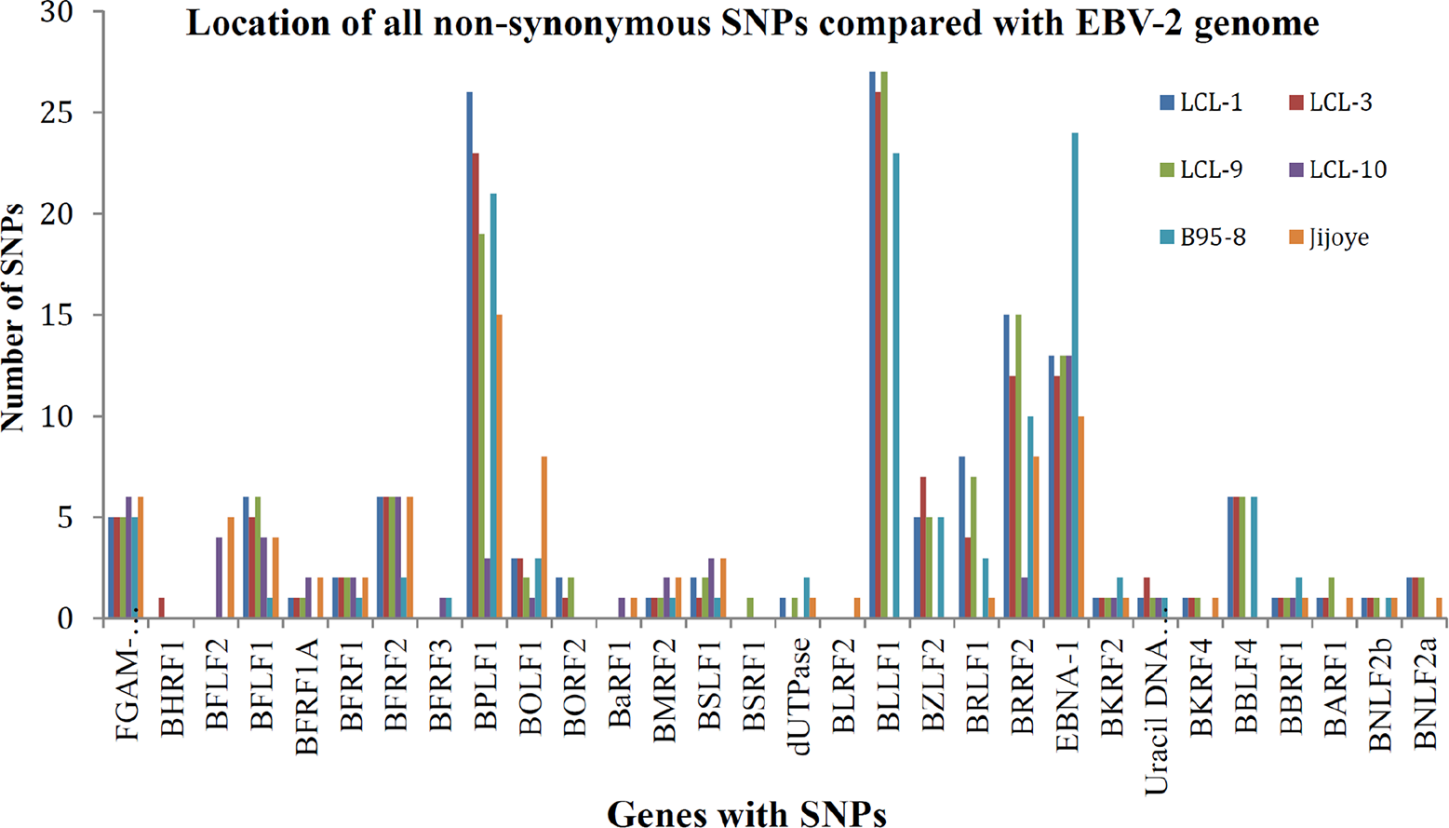
Non—synonymous SNPs compared with EBV-2. We observed that BPLF1, BLLF1, BRRF2, and EBNA1 had the most non—synonymous SNPs compared to EBV-2. For BPLF1 LCL1, LCL3 and B95.8 had the most SNPs; for BLLF1 LCL1, LCL9, LCL3 and B95.8 had the most SNPs, significantly there was no change in LCL-10 in BLLF1; for BRRF2 again most SNPs were noted in LCL1, LCL9, and LCL3; and for latent gene EBNA-1 most SNPs were observed in LCL1, LCL10, LCL9, and B95.8.

**Fig 4:**
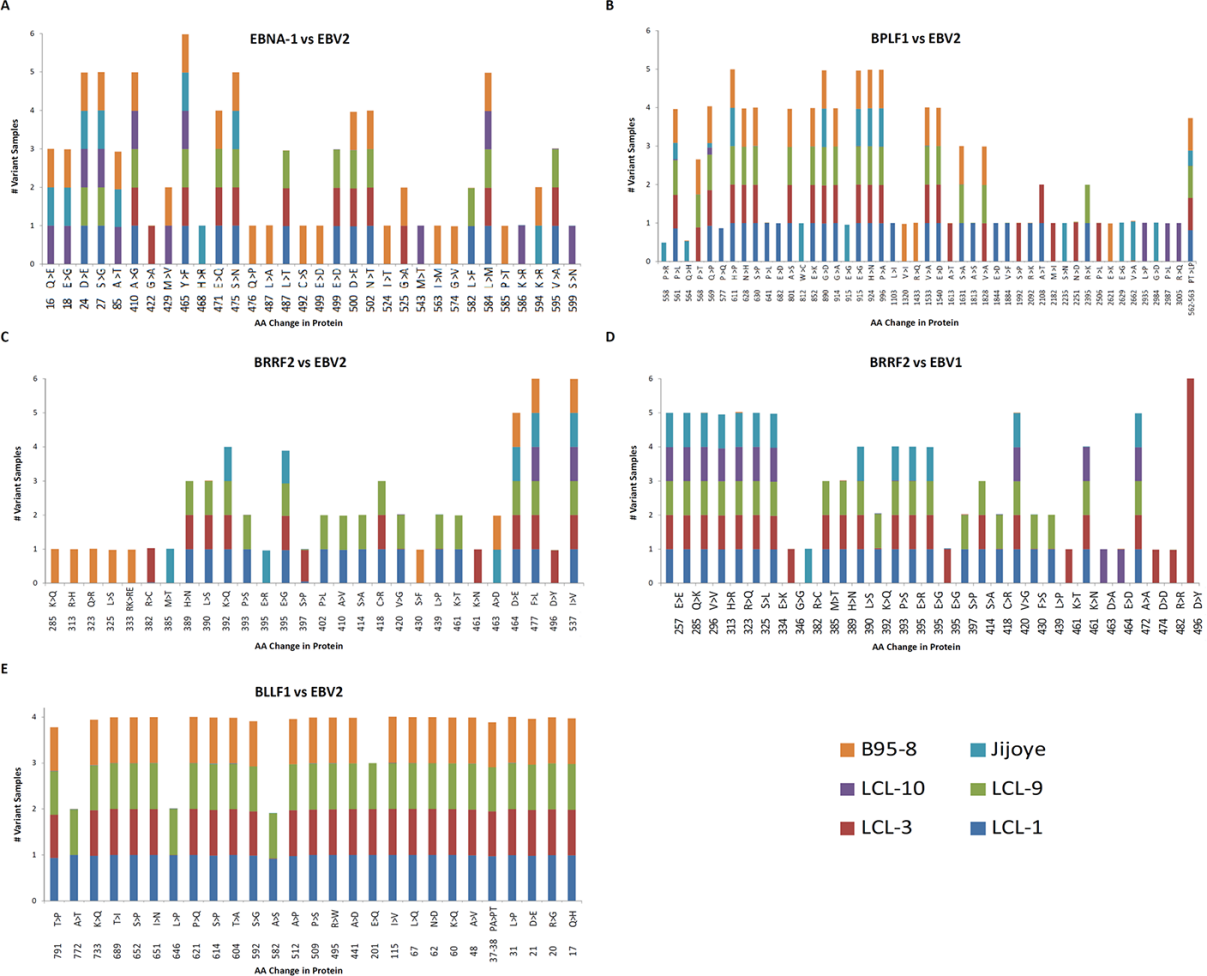
Amino acid changes in EBNA1, BPLF1, BLLF1 and BRRF2. **(A) EBNA1.** This was the only latent gene that had amino acid variation when compared to EBV-2. Towards the N-terminal we observed changes at position 16 and 18 respectively (Q>E, E>G)) for B95.8, Jijoye, and LCL-10, at position 24, 27, 410 (D>E, A>I, A>G) for B95.8, Jijoye, LCL10, LCL9, and LCL1 respectively. At the C-terminal we noted amino acid changes at 543 (M>T), 585 (P>T), and 599 (S>N) for LCL10 alone. **(B) BPLF1.** When we aligned BPLF1 against EBV-2 we noticed several amino acid changes that occurred on specific samples with some toward the N-terminal that were shared by more than 3 samples. The only amino acid changes observed in LCL10 alone occurred at the C-terminal at 2935 (L>P), 2987(P>L), and 3005 (R>Q).**(C) BLLF1.** Comparison of BLLF1 to EBV-2 was interesting in that the amino acid changes were similar in LCL-1, -3, -9, and B95.8, but generally lacking in LCL10 and Jijoye, showing a significant difference between the EBV-1 (B95.8, LCL1,LCL3, LCL9) and EBV-2(LCL10 and Jijoye) clustered samples. **(D) BRRF2.** When BRRF2 was compared to EBV-2 we noted that all the amino acid variations at the N-terminal occurred in B95.8, and in LCL3 at 382 (R>C). Amino acid variations in the middle were mainly in LCL9, LCL3, and LCL1. Amino acid changes at the C-terminal at position 464 (D>E), 477 (F>L), and 537 (I>V) were observed in most of the LCLs, B95.8, and Jijoye. **(E) BRRF2.** On comparing BRRF2 with EBV-1, the only gene we were able to show using Illumina sequencing against EBV-1, we observed that at the N-terminal we had similar amino acid changes in Jijoye, LCL10, LCL9, LCL3, and LCL1. Other amino acid changes occurred in multiple LCLs, except for changes at the C-terminal at positions 463 (D>A) and 464 (E>D) that were both in LCL10. It is to be noted that the amino acid changes in BRRF2 against EBV-2 were significantly different from that observed against EBV-1.

**Fig 5:**
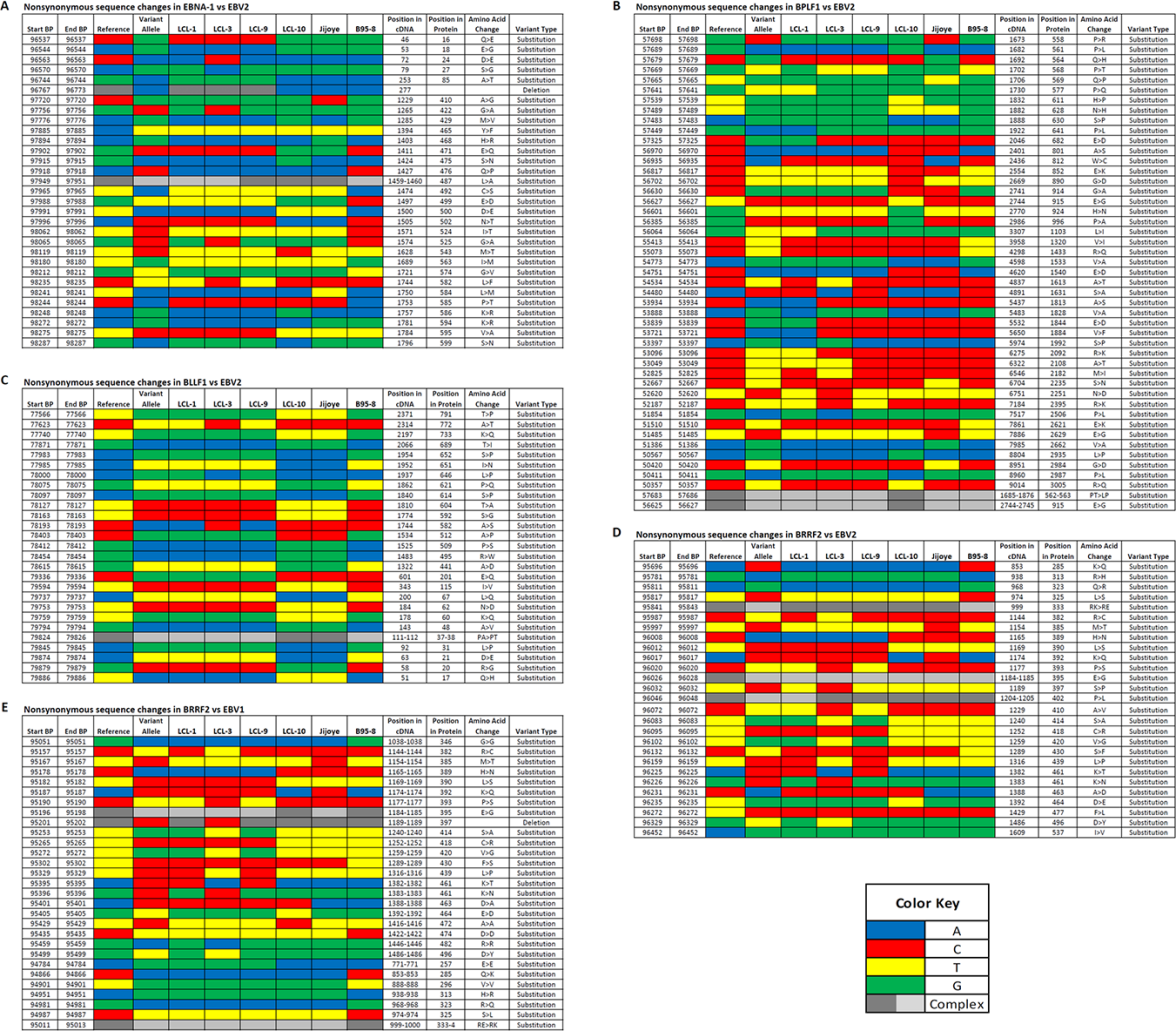
Non-synonymous sequence changes. **(A) EBNA1 compared to EBV-2.** Using heat maps in our analysis we observed that most EBNA1 mutations were substitutions with a single deletion (96733), and that the changes were similar in all LCLs except in a few cases where there were changes in LCL3 (97756) and in LCL10 (98119, 98248, and 98287) distinct from the other LCLs. **(B) BPLF1 compared to EBV-2**. When aligned to EBV-2 all the mutations observed in BPLF1 were substitutions. All the substitutions were generally similar in the LCLs, with some changes seen in LCL3 alone (54534, 53397, 52825, and 51854), LCL10 alone (57689, 57539, 56627, 56385, 50567, 50411, 50357, 57686, and 56 627). **(C) BLLF1 compared to EBV-2**. All the BLLF1 mutations observed were substitutions and they were mostly shared by all LCLs. LCL10 and Jijoye were perfect match with the reference, the rest varied with the reference genome at different sites. LCL1, LCL3, and LCL9 all had similar changes compared to reference except LCL3 at 77623, 78193, and 78000 that were similar to reference. **(D) BRRF2 compared to EBV-2**. We observed that when compared to EBV-2 reference, all the BRRF2 mutations were substitutions, which were similar in most LCLs except for a couple specific for LCL3 (95987 and 96329), and LCL10 (96272). **(E) BRRF2 compared to EBV-1**. When BRRF2 was compared to EBV-1, most of the changes were substitutions, but we had a deletion too that was detected in all samples. Again LCL3 had specific mutations (95157, 95396, 95459, and 95499) and LCL10 (95429). It is to be noted that only LCL3 had no deletion at 95201.

LCL10 derived from a Kenyan child from a malaria holoendemic area was different from the other LCLs that were from malaria hypoendemic region and showed sequence alignment with EBV-2. Besides LCL1, LCL10 had the most non-synonymous SNPs in EBNA1 when aligned against EBV-2. Amino acid changes in the EBNA1 were in position 543 (M>T), 586 (K>R), and 599(S>N) in the C-terminal locus that has been associated with CD4+/CD8+ T cell binding [23, 24]. Significantly, LCL10 had no non-synonymous SNPs in BLLF1, and few in BPLF1 and BRRF2. Further, on comparing LCL10 with Jijoye we noted no significant difference in alignments to EBV-1 and EBV-2 (Table 2), however when we examined the mutations, there were more mutations for Jijoye than LCL10 when compared to either EBV-1 or EBV-2 (Table 3). This was also supported by the observation that the non-synonymous SNP changes in genes were higher for Jijoye than LCL10 when compared to EBV-1 or EBV-2 (Figs 2 and 3). Interestingly, when specific genes were considered, we noted that when EBNA-1 was compared to EBV-2 the amino acid changes for Jijoye were mainly in the N terminus while for LCL10 they were in the N and C terminus (Fig 4A). Similar observation was made for BPLF1 when compared to EBV-2 (Fig 4B). For BLLF1, all the changes were seen in LCLs aligned with EBV-1, but none for LCL10 and Jijoye that aligned with EBV-2 (Fig 4E). Amino acid changes were observed in BRRF2 when compared to either EBV-2 or EBV-1 with changes in EBV-2 significantly different from EBV-1 (Fig 4C and 4D). Using heat maps to compare non-synonymous sequence changes in the genes we again noted that LCL10 was closer to EBV-2 reference than Jijoye, except for BLLF1 in which both were the same as reference EBV-2 (Fig 5C). These observations indicate that LCL10 was closer to EBV-2 than Jijoye, the significance of which warrants further investigation.

### The majority of SNPs in the LCLs were in lytic genes BPLF1, BLLF1, BRRF2, and latent gene EBNA-1

Analysis of the lytic genes in all the LCLs indicated that there were not many non-synonymous SNPs and that most of the changes are those that were inconsequential. Only BLLF1, BPLF1, and BRRF2 posted significant changes in non-synonymous SNPs. Most lytic gene SNPs were conserved in LCL (Figs 2 and 3).

In order to further delineate similarities and differences between the LCLs we looked at base changes and amino acid variations at specific positions in selected genes, EBNA-1 (Figs 4A and 5A), BPLF1 (Figs 4B and 5B), BLLF1 (Figs 4E and 5C), and BRRF2 (Figs 4C and 4D and 5D and 5E) that have been specifically associated with viral latency and entry respectively in the LCL, against reference EBV-2 and EBV-1. Contrary to our expectation of observing similar non-synonymous SNPs for all LCL; when aligned against EBV-2, BPLF1 amino acid changes that affected EBV-1 linked LCLs occurred in the N-terminal, while those affecting LCL10 were at the extreme C-terminal. There were no non-synonymous SNPs and no amino acid variations in BPLF1 when aligned against EBV-1. For BRRF2 amino acid changes at the N-terminal were observed mainly in B95.8 while the other changes were spread across the gene for all the LCLs. Likewise BRRF2 amino acid variation on alignment against EBV-2 were also spread across the gene for all the LCLs. Interestingly, for BLLF1 the amino acid changes were similar for all the LCLs across the gene, but there were no non-synonymous mutations on alignment against EBV-1, and on alignment against EBV-2 the amino acid changes were similar for all LCLs, and B95.8 and Jijoye controls suggesting the significance of the protein in binding to its receptor and conserving the changes perhaps showing the significance of the gene in viral entry into the B cells. These observations indicate how conserved the circulating virus has remained in this geographical region to maintain its transmission. EBNA-1 had most amino acid changes at the N-terminal for EBV-1 linked LCLs and two amino acid changes at the C-terminal for LCL10.

### SNPs in EBV latent genes were observed to be mostly genic

When we analyzed the sequences of the EBV latent genes, the majority of nucleotide changes were genic, intergenic, or synonymous and not predicted to affect function. The changes in LCL10 and Jijoye contrasted with other LCLs when compared to EBV-1 and EBV-2 respectively (Table 3). Analysis of EBNA3A, B, and C against EBV-1 and EBV-2 resulted in all genic and intergenic changes for all LCLs. However, for EBNA-1 gene (and a small potion of EBNA-2 gene), we identified several non-synonymous SNPs when aligned to EBV-2, but not EBV-1 (Fig 4A). The significance of the abundance of genic and intergenic sequences in these latent genes need further study, especially with regard to how they might affect promoters, enhancers and possibly coding and non-coding RNAs.

## Discussion

Previous studies aimed at elucidating EBV-1 and EBV-2 genetic variation in clinical samples have mainly focused on a few short sequences in the latent and lytic genes such as EBNA1, LMP1, LMP2A, and BZLF1 [13]. McGeoch and Gatherer using whole genome sequences compared the latent genes of EBV-1 and EBV-2 looking at SNPs variance [3]. Lin et al. using a *de novo* assembly of genomes from EBV-1 and EBV-2 NGS reads found that the latent genes were unique in defining EBV types and that the BHLF1 and LF3 reading frames were not conserved indicating a non protein-coding function in the EBV life cycle [16]. Kwok et al. used NGS in their study of nasopharyngeal carcinoma biopsy specimen and noted that non-synonymous SNPs were observed mainly in latent, tegument, and glycoprotein genes, as in our LCL samples but did not focus on variation between EBV strains [17]. We have extended the previous studies by using NGS to sequence and analyze the non-repeat regions of the EBV genome in spontaneously derived EBV Type 1 and Type 2 LCL from Kenyan infants.

In this study, using Illumina NGS we were able to classify four LCLs raised from PBMC isolated from Kenyan children. Three of the samples were EBV-1 and one sample was EBV-2. Within these samples and focusing on a few of the genes sequenced, we observed some changes in lytic genes involved in infection of B cells, metabolism of the infected cells, and attachment of the virus among other functions. Among the genes analyzed was BLLF1, an envelop glycoprotein [25] associated with infection of B lymphocytes that has been shown to be mediated by binding of the N-terminal region of the BLLF1 viral glycoprotein to its receptor, CD21, which also serves as the lymphocyte receptor for the C3d molecule, a member of the complement cascade [26]. The BLLF1 viral late glycoprotein, also designated gp350/220 has been shown to be the most abundantly expressed glycoprotein in the viral envelope and to be responsible for stimulating the production of neutralizing antibodies in vivo [27]. Besides mediating adsorption to CD21, BLLF1 glycoprotein binding also induces capping of the receptor and endocytosis of the virus into B lymphocytes [28]. In our study we observed that BLLF1 had the most SNPs resulting in single amino acid and multiple amino acid changes that were in single samples or cluster of samples. The changes were spread throughout the gene and not confined to either the N-terminal or C-terminal. Notably, on alignment against EBV-2 the amino acid changes were similar for LCL1, LCL3, LCL9, and B95.8 that were aligned with EBV-1, while LCL10 and Jijoye control aligned with EBV-2 were similar to EBV-2 reference suggesting the significance of the protein in binding to its receptor and conserving the changes (Figs 4C and 5C).

We further analyzed BRRF2, a tegument protein, whose nucleotide changes were detected with a high frequency in our samples. BRRF2 is a homologue of HSV UL7, which has been associated with extracellular virus, and notably is rarely detected in mature EBV [29]. BRRF2 plays a role in translation [29], viral assembly [30], DNA replication [31], nuclear egress of capsid [32], and has been reported to be involved in multiple other activities such as regulation of transcription [33]. Of interest was the observation that all the amino acid changes at the N-terminal on alignment with EBV-2 were in B95.8, all other amino acid changes were in multiple LCL samples, except for positions 461 (K>N) and 496(D>Y) that both occurred in LCL3 (Fig 4D and 4E) and (Fig 5D and 5E). BRRF2 was the only lytic gene detected with high number of non-synonymous SNPs in both EBV-1 and EBV-2 in our samples (Figs 2 and 3).

BPLF1, whose non-synonymous SNPs were detected in high levels in all the samples encodes a gene transcribed in late lytic phase of EBV replication. BPLF1 protein is a lytic gene product shown to have deubiquitination enzymes (DUB) activity and interacts with TRAF6 to regulate cellular NF-κB signal responses during lytic replication [34–36]. BPLF1 shows homology to HSV1 VP16 and ORF22 of Varicella zoster virus which is a structural tegument protein significant in HSV replication as a trans-activator of viral immediate-early genes [34, 37–41]. BPLF1 was unique in our study as being the gene with the most amino acid changes in specific individual LCL samples, not shared by multiple samples, with most of the changes detected in the C-terminus including those in LCL10 at 2935(L>P), 2987 (P>L), and 3005(R>Q) (Figs 4B and 5B). The significance of these changes warrants further investigation.

EBNA1 is the only EBV latency protein expressed in all EBV associated tumors because of its critical role in initiating EBV episome replication before mitosis [40]. In our LCL samples we noted several non-synonymous SNPs with some affecting only single samples and some in groups of LCLs. Though there were shared amino acid changes among the LCLs, B95.8, and Jijoye at the N-terminal, when aligned against EBV-2 we noted three amino acid changes at the C-terminal at 543 (M>T), 586 (K>R), and 599 (S>N) specific for LCL10 (Figs 4A and 5A). At position 465 (Y>F) the change was common to all samples, including the controls. B95.8 had the most amino acid changes in EBNA-1 with changes mainly in the middle of the gene and towards C-terminal, besides LCL10. When aligned against EBV-1 there was no non-synonymous amino acid changes observed. Paludan et al. have shown that established and TCR-typed EBNA1-specific CD4^+^ T cell clones recognize physiological levels of EBNA1, that are expressed during EBV latency targeting epitopes from the C-terminal [23]. In another study Tsang et al. analyzed the CD4^+^ T-cell responses to EBNA1 in 78 healthy Chinese donors and observed a number of epitopes in the EBNA1 C-terminal region, that included a DP5-restricted epitope that was recognized by almost half of the donors they tested and elicited responses that recognized EBNA1-expressing DP5-positive target cells [24]. We noted that amino acid changes we observed for EBNA1 against EBV-2 were similar at positions 471 (E>Q) for LCL1, 3, 9; 476 (Q>P) for B95.8; 487(L>T) for B95.8, LCL1, -3, 9; 499(E>D) for B95.8, LCL1, 3, 9; 500 (D>E) for B95.8, LCL1, 3, 9; 502 (N>T) for B95.8,-LCL1, 3, 9; 524 (I>T) for B95.8, and 525 (G>A) for B95.8 and LCL3 in EBV wild type, GD1, GD2, and AG876 in an NPC next generation sequencing study by Liu et al. [41, 42]. This maybe a clear case of a recombination process between the African EBV-2 variant and the Asian EBV-1 variant that warrants further investigation.

To make sense of these findings from the small sample size, there is a need to obtain samples from BL patients and healthy controls and sequence EBV from both groups in the same Kenyan geographical area.

Obtaining samples from pediatric subjects is challenging because of the low amount of specimen that can be taken, hence the use of LCL to amplify the EBV genome is one approach to increase the amount of sample and was the method used in our study. The drawback to this method is the likelihood of selecting out a single EBV variant when there could be multiple variants in circulation within a subject. While there was a general concordance between the sequence we obtained for B95-8 genome, there were some differences relative to the published reference sequence of B95-8 [20]. These sequence differences could be attributed to genetic drift from prolonged cell culture in different laboratories [18]. Alternatively, the PCR amplification step could produce point mutations. Though the quality of our samples were good, with high sequencing depth and coverage, it is not inconceivable to have point mutations as a result of the size of the targeted genomic region and the number of samples under analysis. Indeed, we did not observe changes that could be specifically attributed to point mutation affecting a particular LCL or the controls, besides the EBNA1 SNPs that affected all the LCLs and controls leading to amino acid changes [41, 42].

In summary, utilizing the four LCLs to analyze EBV genetic variation, we observed a number of non-synonymous SNPs. We noted conservation in lytic genes in the LCLs as previously reported with respect to EBV-1[16], while nearly all of the changes in latent genes were genic, except for some genetic variation in the EBNA-1 with respect to EBV-2 gene. Some studies have indicated that latent genes are less conserved than lytic genes indicating different evolutionary pressures in the two classes of genes [16, 18, 43]. Interestingly, in our small sample we noted several mutations that caused amino acid changes in several positions in both the lytic and EBNA-1 latent genes when compared to EBV-2, especially LCL1, LCL3, and LCL9 that were closer to EBV-1, however the functional outcome of these changes are currently unknown. Further studies of the LCLs and their sequences may elucidate the significance of the mutations in EBV pathogenesis. Illumina sequencing yielded a robust coverage and mappable reads greater than 90% that could enhance the study of whole EBV genomes from LCLs elucidating variations in lytic and latent genes that may contribute to the EBV-1 and EBV-2 variance and have implications therewith in malignant transformation of infected cells.

## Conclusions

We have shown that genetic variations did occur within the same geographic region in specific genes and that the Illumina MiSeq platform provides a high-throughput sequencing approach that can clearly enhance the study of several EBV genome and other oncogenic viruses in the field, further clarifying the significance of viral strain heterogeneity and geographical distribution in epidemiological studies of the viral initiated cancers.
