# Correction: Epstein-Barr Virus Genetic Variation in Lymphoblastoid Cell Lines Derived from Kenyan Pediatric Population

**DOI:** 10.1371/journal.pone.0130229

**Published:** 2015-06-03

**Authors:** The PLOS ONE Staff

In the Author Contributions section, Eric. E. M. Wohlford should be listed as EMW as being one of the persons who performed the experiments.
